# The Short-Term Outcomes of Intravitreal Faricimab for Treatment-Naïve and -Refractory Neovascular Age-Related Macular Degeneration: A Real-World Study

**DOI:** 10.3390/medicina62050863

**Published:** 2026-04-30

**Authors:** Huai-Lung Chang, Ling-Uei Wang, Tzu-Lun Huang, Pei-Yao Chang, Wei-Ting Ho, Yung-Ray Hsu, Fang-Ting Chen, Yun-Ju Chen, Cheng-Hung (Dixson) Lin, Jia-Kang Wang

**Affiliations:** 1 Department of Ophthalmology, Far Eastern Memorial Hospital, New Taipei City 22060, Taiwan; 2 Department of Medicine, National Taiwan University, Taipei City 106216, Taiwan; 3 Department of Medicine, National Yang Ming Chiao Tung University, Taipei City 112304, Taiwan; 4 Department of Electrical Engineering, Yuan Ze University, Taoyuan City 320315, Taiwan; 5 Department of Electrical Engineering, National Taiwan Normal University, Taipei City 106308, Taiwan

**Keywords:** faricimab, polypoidal choroidal vasculopathy, neovascular age-related macular degeneration

## Abstract

*Background and Objectives*: Neovascular age-related macular degeneration (nAMD), including typical nAMD (tAMD) and polypoidal choroidal vasculopathy (PCV), is a leading cause of visual impairment. This study investigated the real-world short-term outcomes of faricimab, a bispecific antibody targeting Ang-2 and VEGF-A, in patients with treatment-naïve or -refractory nAMD. *Materials and Methods*: This retrospective study analyzed treatment-naïve or -refractory nAMD eyes receiving one, two, or three monthly intravitreal faricimab injections. Primary outcomes were changes in best-corrected visual acuity (BCVA) and central foveal thickness (CFT) one month after the last injection. Secondary outcomes included the dry macula rate (absence of subretinal and intraretinal fluid) and subgroup comparisons between tAMD and PCV. *Results*: After a single injection, both treatment-naïve (*n* = 76) and -refractory (*n* = 44) eyes showed significant CFT reduction (*p* < 0.0001) but no significant BCVA improvement (*p* > 0.05). Dry macula was achieved in 63.2% of treatment-naïve and 71.4% of treatment-refractory eyes. In 38 treatment-naïve eyes receiving three injections, both CFT and BCVA significantly improved from baseline (*p* < 0.001 and *p* = 0.02, respectively), with a 94.7% dry macula rate. Subgroup analysis of those receiving three injections revealed that PCV eyes exhibited significant visual improvement, whereas tAMD eyes did not. No serious systemic or ocular adverse events were observed over the short-term follow-up period. *Conclusions*: Intravitreal faricimab is effective for both treatment-naïve and -refractory nAMD in the short term. While anatomical improvements were comparable between subtypes, the PCV subgroup showed a trend toward greater visual improvement in this small cohort; however, this may be influenced by the significantly younger age of PCV patients. These findings are exploratory and require validation in larger, age-matched prospective studies.

## 1. Introduction

Neovascular age-related macular degeneration (nAMD) is among the leading causes of severe visual impairment worldwide, comprising typical neovascular age-related macular degeneration (tAMD) with macular neovascularization (MNV) and polypoidal choroidal vasculopathy (PCV) with polypoidal lesions [1,2]. Intravitreal anti-vascular endothelial growth factor (VEGF) therapy has been the standard treatment for nAMD [3,4]. Angiopoietin-2 (Ang-2) plays a critical role in vascular destabilization and inflammation regulation, with its expression found to be elevated in nAMD [5,6]. Faricimab, the first bispecific antibody targeting both Ang-2 and VEGF-A, has demonstrated favorable safety and efficacy outcomes in improving best-corrected visual acuity (BCVA) and central foveal thickness (CFT) in the Phase III TENAYA and LUCERNE trials for treatment-naïve nAMD [7,8]. The short-term real-world studies further confirmed its effectiveness and safety for treatment-naïve patients with nAMD [9,10,11,12,13,14,15]. For those patients with nAMD poorly responsive to prior anti-VEGF agents, faricimab also showed treatment value for some of the cases in previous reports [16,17,18,19,20,21,22,23].

The response to anti-VEGF therapy may vary based on different nAMD phenotypes, especially with PCV, a subtype of nAMD. Data from the TENAYA and LUCERNE trials indicate that Asian subgroups are particularly responsive to faricimab management [8]. This efficacy profile appears to extend to specific pathological subtypes, as case series highlight substantial improvements in treatment-naïve patients with PCV, which is more prevalent in Asian populations [8,10,17,18,19,20,21,22,23]. This study aims to evaluate the short-term real-world treatment outcomes of faricimab in treatment-naïve and -refractory nAMD populations, and to compare response patterns in treatment-naïve individuals of PCV and tAMD.

## 2. Materials and Methods

This retrospective study enrolled patients diagnosed with tAMD or PCV from a single center between July 2023 and July 2024. The study was approved by the Institutional Review Board of Far Eastern Memorial Hospital (IRB No. 113303-E) and conducted in accordance with the principles of the Declaration of Helsinki. Informed consent was waived because of the study’s retrospective nature and the fact that the analysis used anonymous clinical data.

### 2.1. Patient Selection

The inclusion criteria were patients who had received at least one faricimab injection for the treatment of tAMD or PCV, with either a naïve or refractory treatment history.

Treatment-naïve eyes were defined as those that had not undergone any prior therapy before faricimab administration. Treatment-refractory eyes were defined as those exhibiting a poor response requiring at least three-monthly injections of prior treatment, with a continued need for treatment intervals of four weeks or less due to persistent fluid or a CFT reduction of less than 50 μm. One or both eyes of each patient were included if they met the diagnostic criteria for tAMD or PCV.

Exclusion criteria included the presence of co-existing retinal diseases in the study eye, optical media opacities compromising image quality, and a history of intraocular surgery in the study eye within three months before or one month after faricimab injection.

### 2.2. Procedure

All participants received at least one intravitreal injection of 0.05 mL faricimab (120 mg/mL; Vabysmo; F. Hoffmann-La Roche Ltd., Kaiseraugst, Switzerland), with some undergoing three monthly injections. The injection procedure was standardized across all cases, including the application of topical proparacaine anesthesia (Alcon, Fort Worth, TX, USA), disinfection with 5% povidone-iodine, and administration of faricimab 3.5 mm from the limbus using a 30-gauge needle.

Post-injection prophylactic topical medications were prescribed four times daily and included one of the following, based on the clinician’s preference: sulfamethoxazole 4% (Taiwan Shionogi & Co., Taipei, Taiwan) for seven days, norfloxacin 0.3% (Sinphar Pharmaceutical Co., Yilan, Taiwan) for three days, gentamicin 0.3% (Sinphar Pharmaceutical Co., Yilan, Taiwan) for seven days, or a combination of tobramycin 0.3% and dexamethasone 0.1% (s.a. Alcon-Couvreur n.v., Bornem, Belgium) for seven days. Follow-up evaluations were scheduled one month after injection for patients receiving a single dose, while those undergoing multiple injections were assessed at one and two months for two injections, or at one, two, and three months following the initial injection for three consecutive doses.

### 2.3. Data Collection

Baseline demographic and clinical data were collected, including age, gender, slit-lamp examination findings, and treatment history (types and number of prior treatments, as well as treatment intervals). BCVA, CFT, the presence of retinal fluid (intraretinal or subretinal) was assessed at baseline and one month after the first injection (P1M). For patients receiving multiple injections, additional data were collected two or three months after the first injection (P2M or P3M, respectively). CFT was defined as the distance between the vitreoretinal surface and the inner surface of the retinal pigment epithelium (RPE), which was done in horizontal and vertical scans and subsequently averaged. If both eyes of the same patient received treatment, then the eye with better baseline BCVA was included.

All OCT images were reviewed by a single investigator. Presentations of pigment epithelial detachment (PED), intraretinal fluid (IRF) and subretinal fluid (SRF) were documented. BCVA was measured using the Early Treatment Diabetic Retinopathy Study (ETDRS) scoring system. Indocyanine green (ICG) angiography was performed on all patients to differentiate between tAMD and PCV.

For treatment-refractory eyes, additional data were collected on the total number of previous intravitreal injections and the types of medications used. Adverse events, including systemic thromboembolic events and serious ocular complications such as intraocular inflammation, new retinal vascular occlusion/vasculitis, retinal detachment, or infectious endophthalmitis, were also recorded.

### 2.4. Outcome Measures

The primary endpoint of the study was the CFT and BCVA changes from baseline to one month after the last faricimab injection. For patients receiving a single injection, this was measured at one-month post-injection (P1M), while for those receiving two or three injections, it was assessed at two (P2M) or three months post-injection (P3M).

Secondary endpoints included the dry macula rate at one month following the final injection, defined as the proportion of eyes with absence of SRF and IRF, as well as the differences in baseline characteristics and treatment outcomes between eyes with tAMD and PCV that received three faricimab injections.

Analyses were conducted separately for treatment-naïve and treatment-refractory eyes. All eyes were further categorized as either tAMD or PCV based on ICG findings.

### 2.5. Statistics

Statistical analyses were performed using GraphPad Prism software (version 7, GraphPad Software Corporation, Boston, MA, USA). Intra-group changes in CFT and BCVA were evaluated using parametric tests (paired *t*-test) when normality could be assumed, while non-parametric tests (Wilcoxon signed-rank test) were applied elsewhere. Comparisons between PCV and tAMD groups were conducted using the non-parametric tests (Mann–Whitney U test).

## 3. Results

A total of 120 patients with 120 eyes completed the one-month follow-up after receiving a single faricimab injection. Among these, 76 eyes were treatment-naïve and 44 eyes were treatment-refractory. Baseline characteristics of treatment-naïve and -refractory patients are presented separately in Table 1. Within the cohort for longitudinal analysis, 63 eyes underwent two-monthly injections (53 treatment-naïve and 10 treatment-refractory), while 43 eyes received three-injections (38 treatment-naïve and 5 treatment-refractory).

### 3.1. CFT and BCVA Changes After One Faricimab Injection

**Figure 1:**
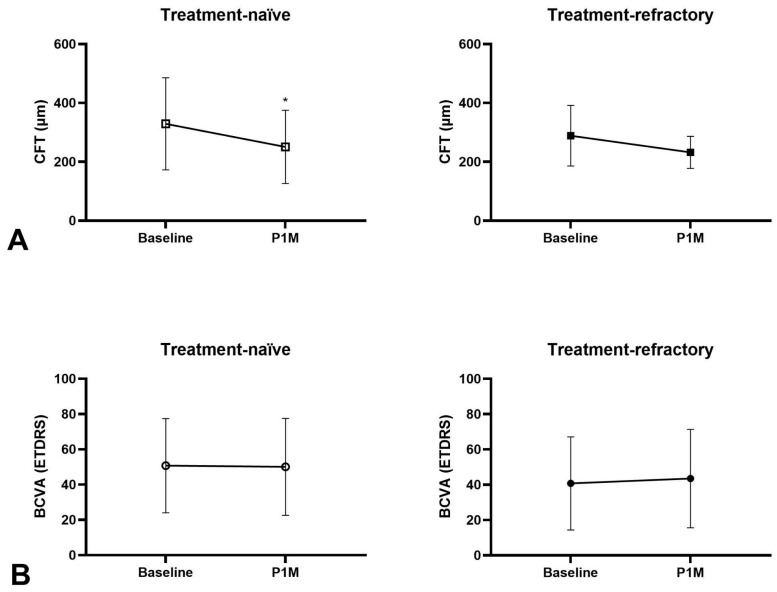
Treatment response for treatment-naïve patients with polypoidal choroidal vasculopathy and typical neovascular age-related macular degeneration receiving one faricimab injection, (**A**) central foveal thickness (CFT) significantly decreased in both treatment-naïve eyes (*p* < 0.00001) and treatment-refractory eyes (*p* < 0.0001) from baseline to one month after first injection of faricimab (P1M). (**B**) Best-corrected visual acuity (BCVA) did not significantly improve in both treatment-naïve eyes (*p* = 0.67) and treatment-refractory eyes (*p* = 0.16) from baseline to P1M. * *p* < 0.05 compared to baseline.

**Figure 2:**
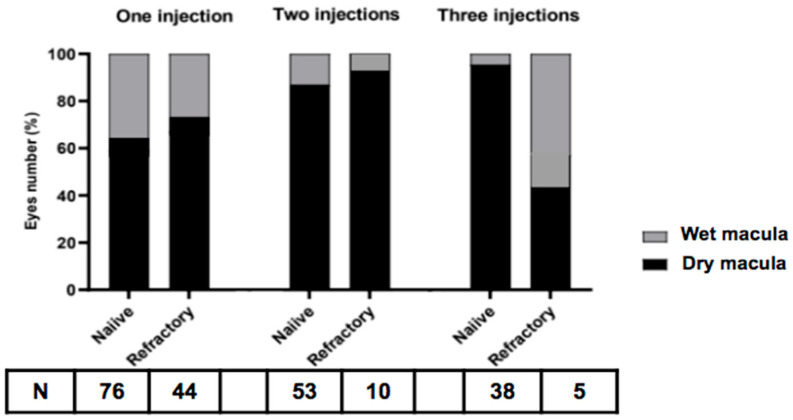
Proportions of dry macula: 63.2% in treatment-naïve eyes and 71.4% in treatment-refractory eyes after one faricimab injection; 84.9% in treatment-naïve eyes and 90.0% in treatment-refractory eyes after two-monthly faricimab injections; 94.7% in treatment-naïve eyes and 40.0% in treatment-refractory eyes after three-monthly faricimab injections.

**Figure 3:**
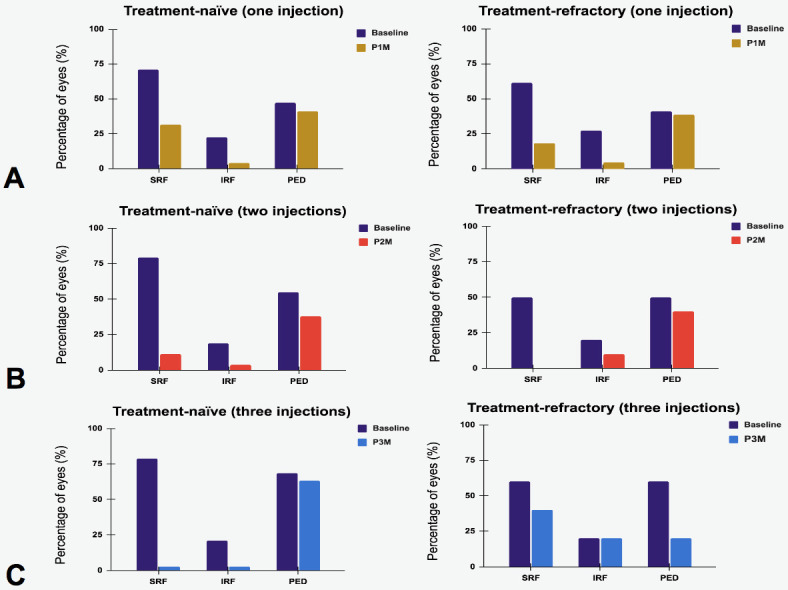
Fluid response to treatment. Presence of intraretinal fluid (IRF), subretinal fluid (SRF), and pigment epithelium detachment (PED) in patients at baseline and after (**A**) single injection of faricimab, (**B**) two-monthly injections of faricimab, and (**C**) three-monthly injections of faricimab.

At one-month post-injection (P1M), treatment-naïve eyes exhibited a mean BCVA decrease of 0.6 letters (50.8 ± 31.9 vs. 50.1 ± 30.1 letters, *p* = 0.67) and a significant mean CFT reduction of 78.5 μm (329.1 ± 156.5 vs. 250.6 ± 146.3 μm, *p* < 0.00001) (Figure 1A,B). Furthermore, 25.0% (19/76) of treatment-naïve eyes achieved a BCVA improvement of two or more lines. The overall dry macula rate was 63.2% among treatment-naïve eyes (Figure 2), including 18.4% resolution of IRF, 39.5% resolution of SRF and 6.6% resolution of PED in treatment-naïve eyes after one faricimab injection (Figure 3A). Subgroup analysis revealed that both tAMD (*N* = 48) and PCV (*N* = 28) eyes demonstrated significant CFT reduction but not statistically significant BCVA improvement. There were no significant differences between tAMD and PCV regarding CFT reduction (82.1 ± 127.7 vs. 72.3 ± 132.2 μm, *p* = 0.78) or BCVA change (−1.40 ± 14.6 vs. +0.57 ± 12.5 letters, *p* = 0.83). No difference in dry macula rate was seen between tAMD and PCV subgroups (68.8% vs. 53.6%, *p* = 0.22).

Treatment-refractory eyes had received an average of 9.98 prior intravitreal injections. The most administered previous treatment was aflibercept (38.9%), followed by ranibizumab (26.7%), brolucizumab (21.1%), and bevacizumab (13.3%). At P1M, these eyes demonstrated a mean BCVA improvement of 2.75 letters (40.8 ± 26.4 vs. 43.5 ± 27.9 letters, *p* = 0.16) and a significant mean CFT reduction of 56.5 μm (288.7 ± 103.1 vs. 232.3 ± 54.3 μm, *p* < 0.0001) (Figure 1A,B). Notably, 25% (11/44) of treatment-refractory eyes achieved a BCVA improvement of two or more lines. The overall dry macula rate among treatment-refractory eyes was 71.4% (Figure 2). There was an 18.4% resolution of IRF, 39.5% resolution of SRF and 2.3% resolution of PED in treatment-refractory eyes after one faricimab injection (Figure 3A). Both tAMD (*N* = 11) and PCV (*N* = 33) eyes exhibited significant CFT reduction but no significant BCVA improvement. There were no significant differences between tAMD and PCV regarding CFT reduction (86.2 ± 93.0 vs. 46.5 ± 74.8 μm, *p* = 0.83) or BCVA change (+1.18 ± 3.49 vs. +3.27 ± 14.6 letters, *p* = 0.56). No difference in dry macula rate was seen between tAMD and PCV subgroups (81.8% vs. 72.7%, *p* = 0.70)

### 3.2. CFT and BCVA Changes After Two-Monthly Faricimab Injections

**Figure 4:**
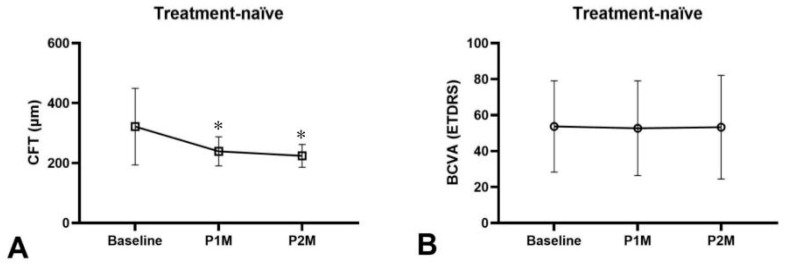
Treatment response for treatment-naïve PCV and tAMD patients receiving two consecutive faricimab treatments. (**A**) Central foveal thickness (CFT) significantly decreased at one month (P1M) and two months (P2M) after injections (*p* < 0.00001). (**B**) Best-corrected visual acuity (BCVA) did not significantly improve at P1M (*p* = 0.53) and P2M (*p* = 0.84). * *p* < 0.05 compared to baseline.

Among 63 eyes that received two consecutive faricimab injections, 53 were treatment-naïve. At one month after the final injection (P2M), treatment-naïve eyes exhibited a nonsignificant mean BCVA decrease of 0.40 letters (53.7 ± 25.4 vs. 53.3 ± 28.8 letters, *p* = 0.84) and a significant mean CFT reduction of 97.3 μm (321.5 ± 127.8 vs. 224.3 ± 37.9 μm, *p* < 0.00001) (Figure 4). Additionally, 24.5% (13/53) of treatment-naïve eyes achieved a BCVA improvement of two or more lines. Overall dry macula rate was 84.9% at P2M (Figure 2), There was a 15.1% resolution of IRF, 67.9% resolution of SRF and 17.0% resolution of PED in treatment-naïve eyes (Figure 3B). There were no significant differences between tAMD and PCV regarding CFT reduction (92.9 ± 121.9 vs. 105.8 ± 185.3 μm, *p* = 0.67) and BCVA change (−2.14 ± 11.0 vs. +3.00 ± 16.9 letters, *p* = 0.07). There was no significant difference in dry macula rate between tAMD and PCV eyes (91.4% vs. 72.2%, *p* = 0.10).

Among the 10 treatment-refractory eyes, compared to baseline at P2M, BCVA increased by 0.10 letters (48.7 ± 25.0 vs. 48.8 ± 28.8 letters, *p* = 0.98), while CFT decreased by 94.4 μm (345.8 ± 140.4 vs. 251.4 ± 57.4 μm, *p* = 0.04). A dry macula was achieved in 90.0% of these eyes (Figure 2). There was a 10.0% resolution of IRF, 50.0% resolution of SRF and 10.0% resolution of PED in treatment-refractory eyes after two-monthly faricimab injections (Figure 3B). Due to the small sample size, subgroup analysis for tAMD and PCV was not conducted.

### 3.3. CFT and BCVA Changes After Three-Monthly Faricimab Injections

**Figure 5:**
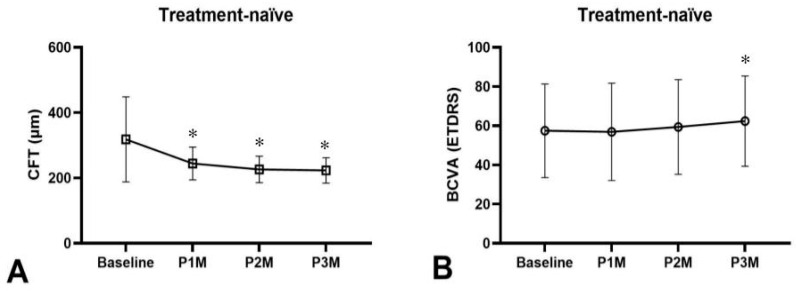
Treatment response for treatment-naïve PCV and tAMD patients receiving three consecutive faricimab treatments. (**A**) CFT significantly decreased at one month (P1M), two months (P2M), and three months (P3M) after injections (*p* < 0.001). (**B**) BCVA did not significantly improve at P1M (*p* = 0.68), P2M (*p* = 0.31), but was significantly better at P3M (*p* = 0.03). * *p* < 0.05 compared to baseline.

**Figure 6:**
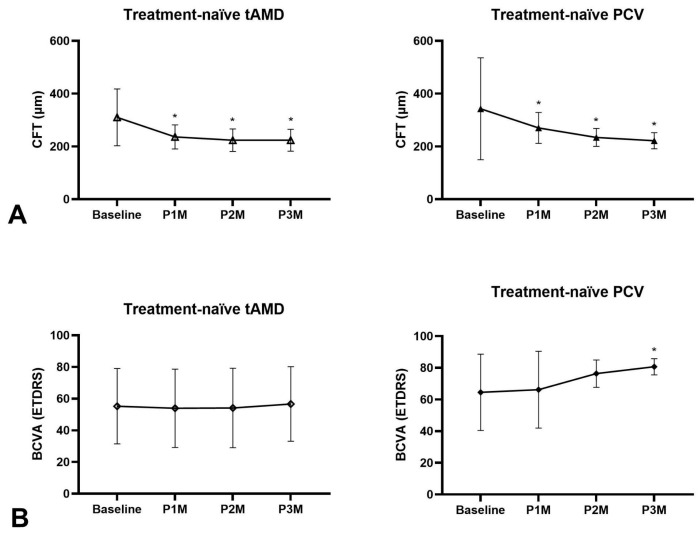
Subgroup analysis of treatment-naïve eyes receiving three consecutive faricimab injections, stratified by diagnosis of tAMD and PCV. (**A**) Both tAMD and PCV subgroups showed significant reductions in CFT from baseline to P1M, P2M, and P3M (*p* < 0.05). (**B**) Significant improvements in BCVA from baseline to P3M were observed only in the PCV subgroup (*p* < 0.05), but not in the tAMD subgroup (*p* > 0.05). * *p* < 0.05 compared to baseline.

Among the 43 eyes that received three consecutive faricimab injections, 38 were treatment-naïve. At one month after the final injection (P3M), treatment-naïve eyes exhibited a significant mean BCVA improvement of 5.6 letters (55.3 ± 26.1 vs. 63.1 ± 23.3 letters, *p* = 0.02) and a significant mean CFT reduction of 94.8 μm (318.0 ± 130.4 vs. 223.2 ± 38.9 μm, *p* < 0.001) (Figure 5). Overall dry macula rate was 94.7% (Figure 2), with only two eyes presenting with wet macula. There was a 18.4% resolution of IRF, 76.3% resolution of SRF and 5.3% resolution of PED in treatment-naïve eyes after three-monthly faricimab injections (Figure 3C). There was no significant dry macula rate difference between tAMD and PCV subgroups (96.6% vs. 72.7%, *p* = 0.42). Both tAMD (*N* = 29) and PCV (*N* = 9) eyes demonstrated significant CFT reductions; however, a statistically significant improvement in BCVA was observed only in PCV eyes from baseline to P3M. There was no significant difference in the magnitude of CFT reduction between tAMD and PCV eyes (86.8 ± 115.5 vs. 120.9 ± 202.8 μm, *p* = 0.92). Notably, PCV eyes exhibited significantly greater BCVA improvement compared to tAMD eyes (+2.39 ± 11.2 vs. +16.1 ± 20.7 letters, *p* < 0.001) (Figure 6A,B). Baseline characteristics of treatment-naïve tAMD and PCV eyes that received three-monthly faricimab injections demonstrated significantly younger age in PCV patients (Table 2).

Among the five treatment-refractory eyes, BCVA increased by 14.6 letters (43.4 ± 33.8 vs. 58.0 ± 16.2 letters, *p* = 0.18), while CFT significantly decreased by 107.8 μm (367.1 ± 169.3 vs. 259.4 ± 42.7 μm, *p* < 0.05). Overall dry macula rate was 40.0% (Figure 2), with three eyes exhibiting persistent fluid on OCT. There was no resolution of IRF, 20.0% resolution of SRF and 40.0% resolution of PED in treatment-refractory eyes after three-monthly faricimab injections (Figure 3C). Given the limited sample size, subgroup analysis for tAMD and PCV was not performed.

### 3.4. Eyes Without Dry Macula After Three-Monthly Faricimab Injections

A total of five out of 43 eyes failed to achieve dry macula despite intensive treatment with three consecutive faricimab injections, including two tAMD eyes and three PCV eyes. Among these, two were treatment-naïve (2/38, 5.3%) and three were treatment-refractory (3/5, 60.0%). Notably, most of these cases were male patients (4/5, 80%), and in one instance, a patient exhibiting a persistent wet macula had a fellow eye that successfully achieved anatomical resolution. Eyes that failed to achieve dry macula after three-monthly faricimab injections were further stratified into eyes with poor response or suboptimal response based on the CFT reduction percentage from baseline to P3M, by CFT decreasing less or more than 10% respectively.

**Figure 7:**
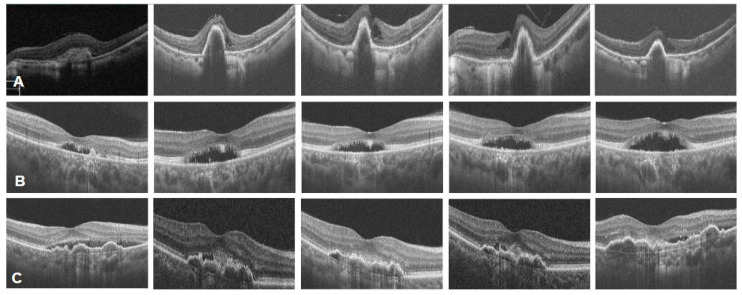
Different response patterns for eyes that failed to achieve dry macula at P3M. (**A**) Delayed response pattern: A 58-year-old female with treatment-naïve tAMD in the left eye. Sequential images (left to right: baseline, P1M, P2M, P3M, and one month post-fifth injection) show persistent IRF and suboptimal CFT reduction from baseline to P3M. Complete macular dryness was eventually achieved only after the fifth injection. (**B**) Persistent non-response pattern: A 47-year-old male with treatment-naïve PCV in the right eye. Sequential images (left to right: baseline, P1M, P2M, P3M, and one month post-fifth injection) reveal refractory SRF and poor CFT reduction that persisted throughout the extended treatment course. (**C**) Tachyphylaxis pattern: A 59-year-old male with treatment-refractory PCV in the right eye. Sequential images (left to right: baseline, P1M, P2M, P3M and one month after a total of 11 injections) demonstrate initial resolution of SRF by P2M, followed by the recurrence of SRF at P3M despite continued treatment.

The two treatment-naïve eyes demonstrated distinct response patterns. One case was a 58-year-old woman with tAMD who exhibited persistent intraretinal cysts and suboptimal CFT reduction (more than 10% CFT reduction compared to baseline) following three injections. Notably, complete fluid resolution was achieved after the fifth injection, suggesting a delayed response (Figure 7A). Conversely, a 47-year-old man with PCV demonstrated non-response, with poor CFT reduction and persistent subretinal fluid despite five injections (Figure 7B).

Among the three treatment-refractory eyes, one 82-year-old male with tAMD had previously received three ranibizumab injections. Suboptimal CFT reduction following three-monthly faricimab injections were perceived, yet eventually failed to achieve dry macula after a total of eight injections. The other two cases, both PCV eyes (one previously received four aflibercept, three ranibizumab and two brolucizumab; the other previously received six aflibercept and two ranibizumab), showed the tachyphylaxis response pattern, with resolution of SRF and/or IRF after two injections, but recurrence of fluid after the third injection. One eye ultimately required 11 faricimab injections to maintain suboptimal response with persistent macula fluid. The other eye switched to aflibercept 8 mg due to inadequate response (Figure 7C).

## 4. Discussion

This study evaluated the anatomical and functional outcomes of faricimab in patients with tAMD and PCV in the real-world setting, stratified by treatment-naïve and treatment-refractory status. Our findings demonstrated that faricimab resulted in significant CFT reduction across all subgroups, with notable visual acuity improvements particularly in PCV patients who received three consecutive injections.

### 4.1. Efficacy of Faricimab in Treatment-Naïve and Treatment-Refractory Eyes

Among treatment-naïve eyes, a single faricimab injection led to a significant reduction in CFT, with a 63.2% dry macula rate after P1M. However, BCVA changes were minimal, suggesting that while anatomical improvements were evident early, functional gains may require sustained treatment. For eyes receiving two injections, dry macula rate increased to 84.9%, though BCVA improvement remained statistically insignificant. In contrast, eyes receiving three consecutive injections experienced both significant CFT reduction and meaningful BCVA improvement, particularly in the PCV subgroup. These findings are consistent with a prior retrospective study reporting significantly better visual outcomes in PCV patients compared to those with tAMD after anti-VEGF treatment [15]. The superior visual acuity response in PCV eyes compared to tAMD may indicate a differential response to faricimab between disease subtypes and patient baseline characteristics; however, the significantly younger age of PCV patients compared to tAMD patients may be a major confounding factor that likely contributed to the more visual recovery observed in the PCV group. Further investigations are warranted to confirm whether PCV patients derive greater functional benefit from faricimab and to assess its long-term efficacy, especially in refractory populations. Furthermore, dry macula rate increased to 94.7% at P3M for those receiving three injections.

For treatment-refractory eyes, faricimab provided anatomical benefits, with significant CFT reduction and dry macula rate of 71.4% after one injection, 90.0% after two injections and 40.0% after three injections. However, BCVA improvements remained modest, likely reflecting chronic structural damage from prior inadequate responses to anti-VEGF therapy. Limited sample size precluded detailed subgroup analysis for PCV and tAMD. These findings suggest that while faricimab offers anatomical benefits for refractory cases, functional improvements may be less predictable and potentially require extended treatment duration.

Our findings corroborate established evidence regarding the efficacy of faricimab in mitigating macular edema and enhancing visual acuity in nAMD. While the pivotal TENAYA and LUCERNE trials utilized a four-dose loading regimen to achieve extended dosing intervals, [8] recent data suggest that a three-monthly loading protocol—as employed in our cohort—yields comparable one-year morphological and functional outcomes for both tAMD and PCV [24]. Real-world evidence further substantiates these trends; the TRUCKEE study demonstrated significant anatomical and functional gains, particularly in treatment-naïve eyes [9]. Notably, substantial reductions in CST were observed after a single injection (−25.3 μM; *p* < 0.001 and a − 84.5 μM; *p* < 0.001 in previously treated and treatment-naïve eyes, respectively), but there was no significant BCVA improvement (+0.7 letters; *p* = 0.196 and a + 4.9 letters; *p* = 0.076 in previously treated and treatment-naïve eyes, respectively) after a single injection of faricimab.

Other retrospective analyses of treatment-naïve tAMD and PCV eyes receiving three-monthly loading doses report consistent visual gains (0.11–0.17 logMAR) and anatomical restoration (mean CFT reduction of 104–181 μM and dry macula rates of 72.3–88.4%) from baseline to three or four months after the first injection [10,12,13,14,15]. Safety profile was favorable with isolated cases of RPE tear and mild inflammation reports by Tanaka et al. and Han et al., respectively. While one study confirmed efficacy across both PCV and type 1/type 2 macular neovascularization [11], most prior real-world studies have not directly compared tAMD and PCV outcomes, a gap our study aims to address.

Recent real-world studies have elucidated the role of faricimab in refractory tAMD and PCV, highlighting primarily anatomical rather than functional benefits. However, interpretation of these findings is complicated by significant methodological heterogeneity; treatment protocols varied from single doses or non-loading regimens [16,19,23] to monthly loading phases [17,20] or mixed approaches [18], with follow-up durations ranging from two to seven months. This variability appears to correlate with the magnitude of anatomical response. For instance, the most intensive protocol—comprising four monthly loading doses—yielded the greatest mean reduction in CFT of 60 µm [20], whereas regimens lacking a loading phase reported no significant CFT improvement [16]. Despite these disparities, a reduction in CFT was a consistent finding across studies (range: 18–60 µm), with dry macula rates ranging from 18% to 39.1% [18,20]. Conversely, functional outcomes were less uniform; BCVA remained largely stable without statistically significant improvement in the majority of cohorts [16,17,18,19,21,22,23]. A notable exception was reported by Aljundi et al., who documented a significant BCVA gain of 0.15 logMAR [20]. Collectively, these data suggest that switching to faricimab is an effective strategy for anatomical restoration in refractory cases, although this does not consistently translate into significant visual recovery in this heavily pre-treated population.

### 4.2. Eyes Without Dry Macula After Three-Monthly Faricimab Injections

Despite overall favorable outcomes, five eyes failed to achieve dry macula following three consecutive faricimab injections. Treatment-refractory eyes had a significantly higher proportion of non-dry macula than treatment-naïve eyes (60.0% and 5.3%, respectively). The persistence of exudative activity in nAMD and PCV eyes despite intensive anti-VEGF therapy, suggests a complex interplay of anatomical and molecular resistance mechanisms. One primary driver of persistent disease activity is macrophage-driven neovascular remodeling, where mature, “arterialized” vessels become less dependent on VEGF for survival and continue to leak despite pharmacological blockade [25]. Furthermore, the upregulation of alternative pro-angiogenic and inflammatory pathways—such as those involving platelet-derived growth factor (PDGF), fibroblast growth factor (FGF), and various chemokines—can bypass the targeted inhibition of VEGF and Ang-2, maintaining pathological angiogenesis [26,27]. In PCV specifically, refractory cases are strongly associated with underlying choroidal structural alterations. The presence of initial pachyvessels (diameter ≥ 180 μm) and a thickened choroid (≥220 μm) are significant predictors of poor anatomical response and recurrent exudation [28]. These pachychoroid-related features suggest that the primary driver in some PCV eyes may be choroidal venous congestion rather than purely VEGF-mediated signaling [28,29]. Additionally, metabolic adaptations such as increased endothelial glycolysis and the development of subretinal fibrosis further contribute to a “recalcitrant” phenotype, where structural damage to the outer retina prevents complete fluid resolution and visual recovery [25,27,30]. Although the small cohort size of resistant cases precluded a robust risk factor analysis, 80% of these cases occurred in male patients. There has been no previously established sex-based risk factor regarding the responsiveness to anti-VEGF agents, but choroidal vasculature and inflammatory processes are evidently different between males and females, [31,32] which might influence disease progression and therapeutic efficacy. These cases underscore the variability in treatment response, particularly in previous treatment-refractory eyes, and highlight the need for further investigation into predictive markers of faricimab efficacy.

This study has some limitations. First, the retrospective design and single-center cohort may limit generalizability. Second, patients completing three injections may represent a more compliant or responsive population, which could influence the more favorable outcomes observed in that group. Third, varying antibiotic regimens introduced uncontrolled variability to the safety results; however, it did not restrict the treatment result from the study. Additionally, the relatively small number of treatment-refractory eyes receiving three injections precluded a robust analysis of this subgroup. Finally, due to the small sample size, our study did not proceed with multivariable analysis of the treatment response in each group to identify the favorable factors, and larger prospective studies are required to perform robust multivariable modeling.

## 5. Conclusions

Faricimab demonstrated significant anatomical benefits in both treatment-naïve and -refractory tAMD/PCV patients, with functional improvements more pronounced in those receiving three consecutive injections. Response rate significantly increased for patients receiving more than one injection. However, a subset of both treatment-naïve and -refractory patients exhibited resistance to faricimab. These findings emphasize the importance of individualized treatment strategies and the need for further research into optimizing faricimab therapy for resistant cases.

## Figures and Tables

**Table 1:** Baseline characteristics of both treatment-naïve and -refractory patients with neovascular age-related macular degeneration.

	Treatment-Naïve (*N* = 76)	Treatment-Refractory (*N* = 44)
Age (years)	70.5 ± 11.3	70.3 ± 9.0
Gender (male:female)	37:39	24:20
tAMD/PCV (n, %)	48 (63.2%)/ 28 (36.8%)	11 (25.0%)/ 33 (75.0%)
Central foveal thickness (μm)	329.1 ± 156.5	288.7 ± 103.1
Best corrected visual acuity (ETDRS letter)	50.8 ± 31.9	40.8 ± 26.4

ETDRS letter: early-treatment of diabetic retinopathy study letters; tAMD: typical neovascular age-related macular degeneration; PCV: polypoidal choroidal vasculopathy.

**Table 2:** Baseline characteristics of treatment-naïve patients with neovascular age-related macular degeneration that received three-monthly faricimab injections.

	Treatment-Naïve tAMD (*N* = 29)	Treatment-Naïve PCV (*N* = 9)	*p* Value
Age (years)	71.6 ± 9.4	61.4 ± 11.5	0.04
Gender (male:female)	12:17	5:4	0.70
Central foveal thickness (μm)	310.4 ± 107.6	342.7 ± 193.0	0.82
Best corrected visual acuity (ETDRS letter)	55.2 ± 23.8	64.6 ± 24.1	0.17

ETDRS letter: early-treatment of diabetic retinopathy study letters; tAMD: typical neovascular age-related macular degeneration; PCV: polypoidal choroidal vasculopathy.

## Data Availability

The data that support the findings of this study are available from the corresponding author, J.-K.W. and L.-U.W., upon reasonable request.
